# Association of Mental Health Services Access and Reincarceration Among Adults Released From Prison in British Columbia, Canada

**DOI:** 10.1001/jamanetworkopen.2022.47146

**Published:** 2022-12-15

**Authors:** Heather Palis, Kevin Hu, William Rioux, Mo Korchinski, Pam Young, Leigh Greiner, Tonia Nicholls, Amanda Slaunwhite

**Affiliations:** 1 BC Centre for Disease Control, Vancouver, British Columbia, Canada; 2 Department of Psychiatry, University of British Columbia, Vancouver, British Columbia, Canada; 3 Unlocking the Gates Services Society, Maple Ridge, British Columbia, Canada; 4 BC Corrections, Victoria, British Columbia, Canada; 5 BC Mental Health and Substance Use Services, Vancouver, British Columbia, Canada; 6 School of Population and Public Health, University of British Columbia, Vancouver, British Columbia, Canada

## Abstract

**Importance:**

Diagnosis of mental disorder is prevalent among people who have been incarcerated. Nevertheless, community mental health services are often limited following release from prison, and reincarceration rates are high. The prevalence of mental disorders is growing among people who are incarcerated in British Columbia (BC), Canada, increasing the urgency of timely and accessible mental health services after release.

**Objective:**

To examine the association of mental health services access and timeliness of services access with reincarceration risk among people released from prison.

**Design, Setting, and Participants:**

In this cohort study, mental disorder diagnoses were derived from *International Classification of Diseases, Ninth Revision* or *International Statistical Classification of Diseases and Related Health Problems, Tenth Revision* codes in administrative health records. Data on prison release and reincarceration were retrieved from corrections records. Population-based health and corrections data were retrieved from the BC Provincial Overdose Cohort, which contains a 20% general population random sample of 1 089 677 BC residents. This study examined releases from provincial prisons between January 1, 2015, and December 31, 2018, among people in the 20% random sample who had a mental disorder diagnosis in the year before their release. Analyses were performed from January to June 2022.

**Exposures:**

Mental health services access (primary care, emergency department visits, or hospitalization) and sociodemographic, health, and incarceration characteristics.

**Main Outcomes and Measures:**

A multistate modeling approach was taken. Cox proportional hazards models were stratified by transition, from release to reincarceration, with and without mental health services access. A state arrival extended model examined the influence of timeliness of mental health services access on subsequent hazard of reincarceration.

**Results:**

A total of 4171 releases among 1664 people (3565 releases among male individuals [84.6%]; 2948 releases [70.7%] among people <40 years old; 2939 releases [70.5%] among people with concurrent substance use disorder diagnosis) were identified. The total study follow-up time was 2834.53 person-years, with a mean (SD) of 0.68 (0.93) years and median (IQR) of 0.25 (0.07-0.84) years per release. Mental health services access was associated with a reduction in the hazard of reincarceration (hazard ratio, 0.61; 95% CI, 0.39-0.94). For each additional month between release and mental health services access, the hazard of reincarceration was increased by 4% (hazard ratio, 1.04; 95% CI, 1.01-1.07).

**Conclusions and Relevance:**

In this cohort study of people with mental disorder diagnoses released from prison in BC, mental health services access was associated with reduced reincarceration risk. These findings suggest that these services may have the greatest impact on reducing reincarceration risk when they are available in a timely manner in the days and weeks immediately following release.

## Key Points

**Question:**

Is access to mental health services associated with reincarceration risk among people released from prison?

**Findings:**

In this cohort study of 1664 adults in British Columbia, Canada, who were released from prison, mental health services access was associated with a significant reduction in the hazard of reincarceration.

**Meaning:**

These findings suggest that the mental health service needs of people released from prison in British Columbia, Canada, remain largely unmet and that timely access to mental health services after release may be critical to reducing reincarceration rates.

## Teaser

This cohort study examines the association of mental health services access and timeliness of services access with reincarceration risk among people released from prison in British Columbia, Canada.

## Introduction

Diagnosis of mental disorder is prevalent among people who have been incarcerated,^1^ and people with mental disorders often live with concurrent conditions, including chronic physical health problems and substance use disorders (SUDs), requiring targeted and individualized care.^2^ Despite high rates of mental health needs, there is considerable evidence of low rates of treatment of mental disorders in correctional settings^3^ and upon release to the community,^4,5^ highlighting the urgency of scaling up interventions to meet these needs.^6^

In Canada, nearly all incarcerated people (>95%) will eventually return to the community.^7,8^ The days and weeks immediately following release represent a time of elevated risk for poor outcomes, such as overdose, recidivism, and death.^9,10,11^ During this period, people who have been incarcerated face a number of barriers to accessing housing, employment, and health services,^12^ a burden that is often compounded among people with mental disorder diagnoses.^13^ These service needs are more widespread now than ever among people who have been incarcerated in British Columbia (BC), given the reported doubling of the prevalence of people with concurrent mental disorders and SUDs between 2009 and 2017 in this population.^14^

People who are incarcerated in provincial prisons in BC have a reincarceration rate of approximately 50% in the 2 years following release.^15^ Although, to our knowledge, this rate has not been explored previously in BC, on the basis of prior studies,^16^ we expect this rate to be even higher for people with mental disorder diagnoses, particularly where other concurrent conditions such as SUDs are present. For example, a recent study^17^ in Indiana found that people with multiple mental health or SUD diagnoses had higher odds of reincarceration. Furthermore, a recent study^18^ conducted in Ontario, Canada, revealed that schizophrenia, bipolar, and personality disorders were associated with elevated rates of return to custody. Studies have suggested that better engagement in treatment for people with mental disorders reduces the risk of recidivism.^16^ For example, routine outpatient treatment has been found to reduce the likelihood of rearrest among people living with severe mental disorders.^19,20^ Re-entry planning and attachment to community resources after incarceration can play a role in strengthening continuity of care and decreasing recidivism. Targeting the period directly following release from custody has been shown to improve contact with community mental health services.^21^ This is particularly relevant for people who have been incarcerated in BC’s provincial prisons, given that sentences are short (an average of 70 days in 2019)^7^ and people often cycle frequently between corrections and community.^4^

These frequent transitions exacerbate existing health, social, and economic inequities faced by people with mental disorder diagnoses. As such, the timeliness of service provision following release is critical to best meet service needs and support safe transitions to community. This cohort study investigates (1) the association of postrelease mental health services access (MHSA) with reincarceration risk and (2) the association of timeliness of MHSA with time to reincarceration. These findings can be used to identify current gaps in the provision of care, highlighting the proportion of people with mental disorder diagnoses for whom mental health service needs remain unmet in the transition from corrections to community.

## Methods

### Study Design, Setting, and Participants

This cohort study used a 20% random sample of the general population of 1 089 677 British Columbians (aged ≥18 years) that is contained within the British Columbia Provincial Overdose Cohort (BC-ODC).^22^ The BC-ODC was created under section 52(2) of British Columbia’s Public Health Act as part of BC’s response to the declaration of the public health overdose emergency in 2016 and brings together administrative health and corrections data. Data in the BC-ODC are linked through BC’s Client Roster in which registration is compulsory in order to access provincial health insurance for all BC residents (including Canadian citizens, permanent residents, and people with visas >6 months and their dependents). Records were linked on a personal identification number assigned by the Ministry of Health. This analysis was conducted using BC-ODC data and was part of the BC Centre for Disease Control’s public health functions; therefore, institutional ethical approval and informed consent were not required. This study follows the Strengthening the Reporting of Observational Studies in Epidemiology (STROBE) reporting guideline.

This analysis included a cohort of people who had a complete record in the client roster between January 1, 2015, and December 31, 2018, including sex, at least 1 release from a provincial correctional center in this period, and a mental disorder diagnosis in the year before release (eFigure 1 in Supplement 1). Mental disorder diagnosis was determined to be present for people with 1 hospitalization or 2 primary care visits with a relevant *International Classification of Diseases*,* Ninth Revision *(*ICD-9*) or *International Statistical Classification of Diseases and Related Health Problems*,* Tenth Revision *(*ICD-10*) code (eTable 1 in Supplement 1) within 1 year of each other, and occurring in the 1 year before their release.^23^ Death data were derived from mortality data from Vital Statistics Agency, which registers all deaths in BC. Eight people who were reincarcerated the day of their release were removed from the analysis.

### Multistate Models

A multistate modeling approach was taken, as is appropriate when the outcome of interest (reincarceration) consists of an intermediate event (MHSA) that is neither an initial nor final state. People with a mental disorder diagnosis who are released from incarceration may be reincarcerated; however, they may or may not access mental health services after release, which may change the risk of reincarceration. In multistate modeling, there is 1 initial state (state 1, release), an intermediate state (state 2, MHSA), and a final state (state 3, reincarceration). The time period for assessment of reincarceration went up to the end of the study period (December 31, 2018) or death.

#### State 1: Release

Release from incarceration was derived from the Ministry of Public Safety and Solicitor General’s records of release from BC’s 10 provincial correctional centers. Each record of release dated between January 1, 2015, and December 31, 2018, was included in the analysis. Analyses were conducted at the release level, and people could contribute multiple releases to the analysis.

#### State 2: Mental Health Services Access

Data on MHSA were retrieved from between January 1, 2015, and December 31, 2018. The exposure was determined at the release level and could change from one release to the next. Visits were determined from* ICD-9* and *ICD-10* codes for mental disorders, derived from primary care, hospitalization, and emergency department records^24,25,26^ (eTable 1 in Supplement 1). The MHSA type is also reported to examine potential differences in the association with reincarceration by service type.

#### State 3: Reincarceration

Reincarceration data were derived from records of admission to and release from provincial correctional centers. For each case of release, a subsequent admission to a provincial correctional center during follow-up was deemed a reincarceration event.

### Measures

Beyond MHSA, other variables determined at the time of each release included age, sex, and health authority of residence (spanning rural and urban geographies across the province) determined from the Client Roster. Ministry of Public Safety and Solicitor General corrections data include records on all of BC’s 10 provincial correctional centers, which span 4 of BC’s 5 regional health authorities (there is no correctional center in BC’s most urban health authority). Corrections variables recorded include length of most recent incarceration and year of release.

Co-occurring SUD was defined as the presence of 1 hospitalization record or 2 outpatient records identified within 1 year with *ICD-9* or *ICD-10* codes for SUD^23^ (eTable 2 in Supplement 1). Only those who were determined to have a SUD in the year before release were considered, to capture recent SUD. The Elixhauser comorbidity index was derived, removing the 3 disease groups reflecting SUDs and mental disorders,^27^ presented as a 3-level categorical variable reflecting the number of comorbidities present (0, 1, or ≥2) (eTable 3 in Supplement 1). Social assistance was derived from Ministry of Social Development and Poverty Reduction records of unemployment and disability payments. People who received 1 or more payments in the year before their release were considered to have received a recent social assistance payment.

### Statistical Analysis

#### Estimation

To prepare the data, a time variable (days since release) and a binary indicator (happened or not) were required for both MHSA (state 2) and reincarceration (state 3). For those who did not enter state 2 or 3, time was the end of study period or death date, whichever came first.

Covariate effects were estimated by a set of Cox proportional hazard models stratified by transitions. The correlation between releases from the same person was handled by computing robust variance to account for the clustered data, deemed to be equivalent to generalized estimation equation approaches.^28^ Proportional hazards were assumed for the 2 transitions with the same receiving state (from state 1 to state 3, and from state 2 to state 3). These transitions were considered as part of the same stratum (receiving state 3), and transition from state 1 to state 2 belonged to its own stratum (receiving state 2). A new binary indicator called MHSA was created to distinguish whether mental health services were accessed in those in the receiving state 3 stratum, to differentiate the transition from state 1 to state 3 (MHSA = 0) and from state 2 to state 3 (MHSA = 1). This indicator was included in the model to estimate the association of MHSA with reincarceration under the proportional hazards assumption.

#### State Arrival Extended Model

State arrival time was added to the stratified Cox proportional hazards models as a covariate to examine the influence of the timeliness of MHSA (time at which person arrived in state 2) on the subsequent transition hazard of reincarceration. The unit of time variable was aggregated to month (rather than day) to observe a larger hazard ratio (HR) for a 1-unit increase in the state arrival time. Function forms for time were tested, to check the linearity between MHSA time and hazards of reincarceration using a smoothing spline (eFigure 2, eFigure 3, and eFigure 4 in Supplement 1). There were no missing data on any of the variables included in the analysis. Analyses were conducted to report on reincarceration hazard with and without MHSA, by mental disorder type, and by SUD diagnoses. Plots of the probability of being at each state over time (state occupation probability plots) were generated to demonstrate the probability of reincarceration for hypothetical participants, holding covariates constant (eFigure 5 and eFigure 6 in Supplement 1). We considered results as statistically significant if the 95% CI did not contain 1 or if *P* < .05 (1-sided Pearson χ^2^ test in Table 1). Analyses were performed between January and June 2022 using the survival and mstate packages in R statistical software version 3.5.2 (R Project for Statistical Computing).

**Table 1.** Characteristics of the Sample by Transition

Characteristic	Total releases, No. (%) (N = 4171)^a^	Release to reincarceration, No. (%) (N = 4171)^b^			Release to mental health services, No. (%) (N = 4171)^b^			Mental health services to reincarceration, No. (%) (n = 1927)^b^			
		No (n = 2478)	Yes (n = 1693)	*P* value^c^	No (n = 2244)	Yes (n = 1927)	*P* value^c^	Overall (n = 1927)	No (n = 785)	Yes (n = 1142)	*P* value^c^
Age, y											
<30	1383 (33.2)	756 (30.5)	627 (37.0)	<.001	805 (35.9)	578 (30.0)	<.001	578 (30.0)	206 (26.2)	372 (32.6)	<.001
30-39	1565 (37.5)	926 (37.4)	639 (37.7)		845 (37.7)	720 (37.4)		720 (37.4)	287 (36.6)	433 (37.9)	
40-49	836 (20.0)	523 (21.1)	313 (18.5)		426 (19.0)	410 (21.3)		410 (21.3)	169 (21.5)	241 (21.1)	
≥50	387 (9.3)	273 (11.0)	114 (6.7)		168 (7.5)	219 (11.4)		219 (11.4)	123 (15.7)	96 (8.4)	
Sex											
Female	606 (14.5)	381 (15.4)	225 (13.3)	.06	299 (13.3)	307 (15.9)	.02	307 (15.9)	139 (17.7)	168 (14.7)	.08
Male	3565 (85.5)	2097 (84.6)	1468 (86.7)		1945 (86.7)	1620 (84.1)		1620 (84.1)	646 (82.3)	974 (85.3)	
Health authority											
Fraser	1323 (31.7)	802 (32.4)	521 (30.8)	<.001	629 (28.0)	694 (36.0)	<.001	694 (36.0)	284 (36.2)	410 (35.9)	<.001
Interior	444 (10.6)	329 (13.3)	115 (6.8)		162 (7.2)	282 (14.6)		282 (14.6)	113 (14.4)	169 (14.8)	
Northern	260 (6.2)	161 (6.5)	99 (5.8)		123 (5.5)	137 (7.1)		137 (7.1)	51 (6.5)	86 (7.5)	
Vancouver Coastal Health	861 (20.6)	545 (22.0)	316 (18.7)		376 (16.8)	485 (25.2)		485 (25.2)	166 (21.1)	319 (27.9)	
Vancouver Island	518 (12.4)	323 (13.0)	195 (11.5)		233 (10.4)	285 (14.8)		285 (14.8)	130 (16.6)	155 (13.6)	
Unknown	765 (18.3)	318 (12.8)	447 (26.4)		721 (32.1)	44 (2.3)		44 (2.3)	41 (5.2)	3 (0.3)	
Elixhauser comorbidity index											
0	3501 (83.9)	2032 (82.0)	1469 (86.8)	<.001	1971 (87.8)	1530 (79.4)	<.001	1530 (79.4)	645 (82.2)	885 (77.5)	<.001
1	456 (10.9)	302 (12.2)	154 (9.1)		186 (8.3)	270 (14.0)		270 (14.0)	79 (10.1)	191 (16.7)	
≥2	214 (5.1)	144 (5.8)	70 (4.1)		87 (3.9)	127 (6.6)		127 (6.6)	61 (7.8)	66 (5.8)	
Social assistance											
No	2273 (54.5)	1325 (53.5)	948 (56.0)	.11	1404 (62.6)	869 (45.1)	<.001	869 (45.1)	433 (55.2)	436 (38.2)	<.001
Yes	1898 (45.5)	1153 (46.5)	745 (44.0)		840 (37.4)	1058 (54.9)		1058 (54.9)	352 (44.8)	706 (61.8)	
Concurrent substance use disorder											
No	1232 (29.5)	764 (30.8)	468 (27.6)	.03	731 (32.6)	501 (26.0)	<.001	501 (26.0)	263 (33.5)	238 (20.8)	<.001
Yes	2939 (70.5)	1714 (69.2)	1225 (72.4)		1513 (67.4)	1426 (74.0)		1426 (74.0)	522 (66.5)	904 (79.2)	
Length of most recent incarceration, d											
<4	902 (21.6)	545 (22.0)	357 (21.1)	.009	426 (19.0)	476 (24.7)	<.001	476 (24.7)	209 (27.1)	267 (23.4)	.03
4-15	1184 (28.4)	672 (27.1)	512 (30.2)		614 (27.4)	570 (29.6)		570 (29.6)	213 (27.1)	357 (31.3)	
16-60	1267 (30.4)	738 (29.8)	529 (31.2)		679 (30.3)	588 (30.5)		588 (30.5)	228 (29.0)	360 (31.5)	
>60	818 (19.6)	523 (21.1)	295 (17.4)		525 (23.4)	293 (15.2)		293 (15.2)	135 (17.2)	158 (13.8)	
Year of release											
2015	982 (23.5)	545 (22.0)	437 (25.8)	<.001	485 (21.6)	497 (25.8)	<.001	497 (25.8)	152 (19.4)	345 (30.2)	<.001
2016	1167 (28.0)	663 (26.8)	504 (29.8)		605 (27.0)	562 (29.2)		562 (29.2)	189 (24.1)	373 (32.7)	
2017	976 (23.4)	565 (22.8)	411 (24.3)		505 (22.5)	471 (24.4)		471 (24.4)	189 (24.1)	282 (24.7)	
2018	1046 (25.1)	705 (28.5)	341 (20.1)		649 (28.9)	397 (20.6)		397 (20.6)	255 (32.5)	142 (12.4)	
Mental health diagnosis											
Schizophrenia and other psychotic disorders	923 (22.1)	553 (22.3)	370 (21.9)	.30	457 (20.4)	466 (24.2)	.004	466 (24.2)	164 (20.9)	302 (26.4)	.04
Mood disorders	1113 (26.7)	685 (27.6)	428 (25.3)		592 (26.4)	521 (27.0)		521 (27.0)	231 (29.4)	290 (25.4)	
Stress-related disorders	1050 (25.2)	603 (24.3)	447 (26.4)		592 (26.4)	458 (23.8)		458 (23.8)	185 (23.6)	273 (23.9)	
Personality and behavioral disorders	494 (11.8)	283 (11.4)	211 (12.5)		291 (13.0)	203 (10.5)		203 (10.5)	82 (10.4)	121 (10.6)	
Other disorders^d^	591 (14.2)	354 (14.3)	237 (14.0)		312 (13.9)	279 (14.5)		279 (14.5)	123 (15.7)	156 (13.7)	
Time to service access, mean (SD), mo	NA	NA	NA	NA	NA	NA	NA	1.9 (3.7)	2.8 (5.0)	1.2 (2.3)	
Mental health service access type											
Outpatient primary care	NA	NA	NA	NA	NA	NA	NA	966 (50.1)	417 (53.1)	549 (48.1)	.002
Outpatient emergency care	NA	NA	NA	NA	NA	NA	NA	138 (7.2)	35 (4.5)	103 (9.0)	
Counseling	NA	NA	NA	NA	NA	NA	NA	331 (17.2)	147 (18.7)	184 (16.1)	
Outpatient specialist care	NA	NA	NA	NA	NA	NA	NA	135 (7.0)	56 (7.1)	79 (6.9)	
Hospitalization	NA	NA	NA	NA	NA	NA	NA	177 (9.2)	62 (7.9)	115 (10.1)	
Emergency department visit	NA	NA	NA	NA	NA	NA	NA	67 (3.5)	25 (3.2)	42 (3.7)	
Other^e^	NA	NA	NA	NA	NA	NA	NA	113 (5.9)	43 (5.5)	70 (6.1)	

Abbreviation: NA, not applicable.

^a^ Transitions from state 1 to state 2 and from state 1 to state 3 have the same overall number of releases (4171 releases; starting state is 1, release), whereas transition from state 2 to state 3 has a smaller number of releases (1927 releases; starting state 2, mental health services access), because the starting state is those who had accessed mental health services.

^b^ Among those who were ever reincarcerated, there were a median of 2 reincarcerations per person (range, 1-53 reincarcerations per person; IQR, 1-3 reincarcerations per person).

^c^ Calculated with Pearson χ^2^ test.

^d^ Other disorders includes neurocognitive disorders, intellectual disabilities, disorders of psychological development, and multiple, other or unspecified mental disorders. See eTable 1 in Supplement 1 for further information.

^e^ Other refers to all services with a frequency of 50 or less and includes miscellaneous and other visits (10 releases), institutional visits (17 releases), no charge referral (6 releases), consultation (3 releases), minor surgery or other procedure (5 releases), home visits (6 releases), other unspecified encounters (50 releases), visit premiums (13 releases), and pathology (3 releases).

#### Sensitivity Analysis

The state arrival extended model was compared with a stratified model to verify that the proportional hazards assumption was met. In the stratified model, separate baseline hazards were determined for each transition. The hazard curves of the 2 transitions to reincarceration were similar in shape, indicating that the assumption of proportional hazards was reasonable (eFigure 7 in Supplement 1). We also examined the reincarceration rate for people with and without a mental disorder diagnosis in the year before their release (eTable 4 in Supplement 1) and examined the association of SUD services access after release and reincarceration (eTable 5 in Supplement 1).

## Results

**Figure 1.**
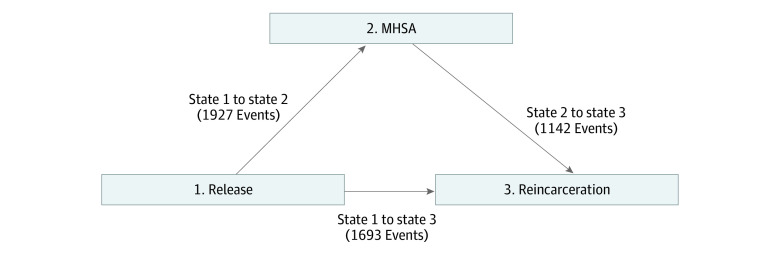
Multistate Model of Release, Mental Health Services Access (MHSA), and Reincarceration Of the 4171 releases, 1693 (40.6%) transitioned from release to reincarceration without MHSA (state 1 to state 3), and 1927 (46.2%) transitioned from release to MHSA (state 1 to state 2); of these, 1142 releases (59.3%) were reincarcerated during follow-up, after their MHSA (state 2 to state 3). The remaining 551 releases did not have MHSA, nor was reincarceration observed during follow-up.

The sample included 4171 releases among 1664 people (3565 releases among male individuals [84.6%]; 2948 releases [70.7%] among people <40 years old; 2939 releases [70.5%] among people with concurrent SUD diagnosis) (eFigure 1 in Supplement 1). The total study follow-up time was 2834.53 person-years, with a mean (SD) of 0.68 (0.93) years and median (IQR) of 0.25 (0.07-0.84) years per release. The multistate model grouped the 4171 releases into 3 states, according to status of MHSA and reincarceration. Of the 4171 releases, 40.6% (1693 releases) transitioned from release to reincarceration without MHSA (state 1 to state 3), 46.2% (1927 releases) transitioned from release to MHSA (state 1 to state 2), and of these, 59.3% (1142 releases) were reincarcerated during follow-up, after their MHSA (state 2 to state 3) (Figure 1).

Characteristics of releases that did and did not make each transition are reported in Table 1. These 3 transitions formed the foundation of the subsequent Cox proportional hazard models, whereby HRs for a number of variables are reported in relation to each of the 3 transitions (Table 2). The estimate of the overall association of MHSA with reincarceration revealed that MHSA was associated with a reduction in the hazard of reincarceration (HR, 0.61; 95% CI, 0.39-0.94). State arrival time was added to the model to determine the association of time between release and MHSA (state 1 to state 2) and hazard of reincarceration. This estimate was statistically significant and indicated that for each additional month of time that passed between release and MHSA, the hazard of reincarceration was increased by 4% (HR, 1.04; 95% CI, 1.01-1.07).

**Table 2.** HRs for Each of the 3 Transitions Estimated From Stratified Cox Proportional Hazards Models

Variable	HR (95% CI)		
	Release to reincarceration (state 1 to state 3)	Release to mental health services (state 1 to state 2)	Mental health services to reincarceration (state 2 to state 3)
Receiving state stratum^a^	State 3	State 2	State 3
Age group, y			
<30	1 [Reference]	1 [Reference]	1 [Reference]
30-39	0.87 (0.75-1.01)	0.98 (0.85-1.14)	0.94 (0.77-1.15)
40-49	0.81 (0.67-0.98)	0.95 (0.80-1.12)	0.77 (0.63-0.95)
≥50	0.62 (0.48-0.80)	1.02 (0.83-1.26)	0.55 (0.41-0.75)
Sex			
Female	1 [Reference]	1 [Reference]	1 [Reference]
Male	1.37 (1.14-1.65)	1.00 (0.86-1.16)	1.25 (1.04-1.51)
Health authority			
Fraser	1 [Reference]	1 [Reference]	1 [Reference]
Interior	0.62 (0.48-0.80)	1.14 (0.94-1.37)	1.07 (0.85-1.34)
Northern	0.97 (0.76-1.13)	0.93 (0.76-1.15)	1.07 (0.81-1.43)
Vancouver Coastal	0.92 (0.76-1.13)	1.07 (0.90-1.27)	1.18 (0.92-1.51)
Vancouver Island	0.88 (0.71-1.10)	0.98 (0.82-1.18)	0.93 (0.74-1.16)
Unknown	1.29 (1.03-1.63)	0.07 (0.05-1.10)	0.25 (0.08-0.78)
Elixhauser comorbidity index			
0	1 [Reference]	1 [Reference]	1 [Reference]
1	1.17 (0.88-1.55)	1.33 (1.06-1.66)	1.31 (0.94-1.82)
≥2	0.87 (0.62-1.22)	1.02 (0.83-1.25)	0.85 (0.63-1.14)
Social assistance in prior year			
No	1 [Reference]	1 [Reference]	1 [Reference]
Yes	1.36 (1.14-1.62)	1.17 (1.03-1.32)	1.29 (1.11-1.50)
Substance use disorder in prior year			
No	1 [Reference]	1 [Reference]	1 [Reference]
Yes	1.53 (1.32-1.78)	1.02 (0.92-1.16)	1.32 (1.13-1.55)
Sentence length, d			
<4	1 [Reference]	1 [Reference]	1 [Reference]
4-15	1.03 (0.90-1.18)	0.91 (0.80-1.02)	1.17 (0.97-1.42)
16-60	0.93 (0.80-1.08)	0.96 (0.84-1.08)	1.10 (0.92-1.32)
>60	0.49 (0.41-0.59)	0.87 (0.75-1.02)	0.94 (0.76-1.15)
Release year			
2015	1 [Reference]	1 [Reference]	1 [Reference]
2016	0.94 (0.81-1.08)	0.93 (0.81-1.06)	1.04 (0.88-1.23)
2017	0.89 (0.76-1.05)	0.90 (0.78-1.03)	1.01 (0.84-1.22)
2018	0.81 (0.68-0.96)	0.89 (0.77-1.02)	0.80 (0.62-1.02)
Mental health diagnosis			
Schizophrenia	1 [Reference]	1 [Reference]	1 [Reference]
Mood disorder	0.72 (0.60-0.86)	0.83 (0.71-0.97)	0.96 (0.80-1.17)
Stress-related disorder	0.72 (0.60-0.85)	0.61 (0.53-0.72)	0.97 (0.79-1.19)
Personality and behavioral disorders	0.81 (0.65-1.02)	0.75 (0.62-0.91)	1.13 (0.88-1.44)
Other disorders^b^	0.78 (0.62-0.98)	0.73 (0.61-0.87)	0.94 (0.77-1.14)
Mental health services access, yes^c^	NA	NA	0.61 (0.39-0.94)
Time to mental health services access, mo^c^	NA	NA	1.04 (1.01-1.07)
Mental health services access type			
Outpatient primary care	1 [Reference]	1 [Reference]	1 [Reference]
Outpatient emergency care	NA	NA	1.41 (1.08-1.83)
Counseling	NA	NA	0.91 (0.75-1.11)
Outpatient specialist care	NA	NA	1.10 (0.84-1.43)
Hospitalization	NA	NA	1.15 (0.90-1.46)
Emergency department visit	NA	NA	1.21 (0.83-1.78)
Other^d^	NA	NA	1.41 (1.06-1.87)

Abbreviations: HR, hazard ratio; NA, not applicable.

^a^ Transitions from state 1 to state 3 and from state 2 to state 3 are in 1 stratum and share a common baseline hazard (receiving state 3). The transition from state 1 to state 2 is in a separate stratum (receiving state 2).

^b^ Other disorders includes neurocognitive disorders, intellectual disabilities, disorders of psychological development, and multiple, other or unspecified mental disorders. See eTable 1 in Supplement 1 for further information.

^c^ The association of mental health access and timeliness with the outcome are estimated to be HR = 0.61 and HR = 1.04, respectively.

^d^ Other refers to all services with a frequency of 50 or less and includes miscellaneous and other visits (10 releases), institutional visits (17 releases), no charge referral (6 releases), consultation (3 releases), minor surgery or other procedure (5 releases), home visits (6 releases), other unspecified encounters (50 releases), visit premiums (13 releases), and pathology (3 releases).

Overall, older age was associated with a lower hazard of reincarceration, both in the presence and absence of MHSA. Male participants had a higher hazard of reincarceration in the absence (HR, 1.37; 95% CI, 1.14-1.65) and presence (HR, 1.25; 95% CI, 1.04-1.51) of MHSA. A concurrent SUD diagnosis was associated with a higher hazard of reincarceration, in the absence (HR, 1.53; 95% 1.32-1.78) and presence (HR, 1.32 (95% CI, 1.13-1.55) of MHSA. Time in custody longer than 60 days was associated with less than half the hazard of reincarceration (HR, 0.49; 95% CI, 0.41-0.59) in the transition directly from release to reincarceration compared with a length of stay in custody less than 4 days. Releases in 2018 had a 20% reduced hazard of reincarceration directly following release (state 1 to state 3) compared with releases in 2015.

Mental disorder diagnosis type revealed significant differences in terms of hazards. For example, all mental disorder types were associated with lower hazards of MHSA (state 1 to state 2) compared with schizophrenia and other psychotic disorders. Furthermore, mood disorders, stress disorders, and other disorders were all associated with a significantly lower hazard of reincarceration directly following release (state 1 to state 3) compared with schizophrenia and other psychotic disorders. Interestingly, there were no significant differences in the hazard of reincarceration by mental disorder type in the presence of MHSA (state 2 to state 3). When considering MHSA type, the hazard of reincarceration was significantly higher for emergency outpatient care visits (HR, 1.41; 95% 1.08-1.83) and other visits (HR, 1.41; 95% 1.06-1.87) compared with outpatient primary care visits.

**Figure 2.**
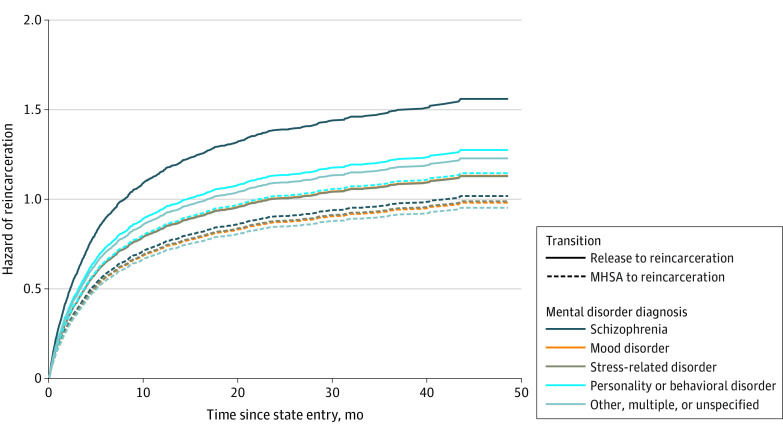
Hazard Estimates by Transition and Mental Health Diagnosis Type Mental health service access (MHSA) time is assumed at the mean time for those who accessed services (1.87 months after release). Other disorders refers to neurocognitive disorders, intellectual disabilities, disorders of psychological development, and multiple, other, or unspecified mental disorders (ie, where disorder type was not specified by billing physician). See eTable 1 in Supplement 1 for further information.

**Figure 3.**
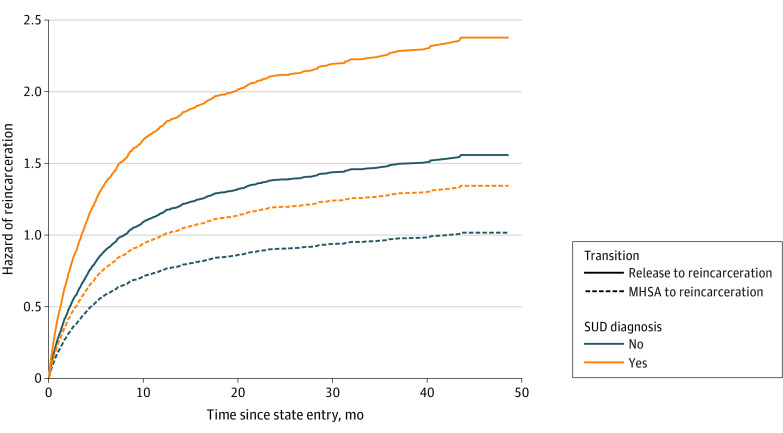
Hazard Estimates by Transition and Substance Use Disorder (SUD) Diagnosis Mental health service access (MHSA) time is assumed at the mean time for those who accessed services (1.87 months after release).

The hazard of reincarceration is reported by transition and mental disorder types (Figure 2). The schizophrenia and other psychotic disorders group had the highest hazard of reincarceration, compared with all other mental disorder types. For all mental disorder types, the hazard of reincarceration was lower in the presence of MHSA. In sensitivity analyses, we confirmed that the reincarceration rate was higher among people with a mental disorder diagnosis in the year before release, compared with people without a mental disorder diagnosis (68.1% [1134 releases] vs 40.5% [1632 releases]; χ^2^_2_ = 364.68; *P* < .001) (eTable 4 in Supplement 1). When considering the hazard of reincarceration by transition and SUD diagnosis (Figure 3), there was an association between MHSA and reincarceration among people with and without SUD diagnoses. This association appeared to be greater in the SUD diagnosis group, for whom the gap between hazard of reincarceration for those with and without MHSA was larger than that observed in the no SUD group. Sensitivity analysis conducted among people with SUD revealed that the risk of reincarceration was lower where SUD services were accessed after release, in the presence (HR, 0.46; 95% CI, 0.39-0.56) and absence (HR, 0.33; 95% CI, 0.29-0.37) of MHSA after release (eTable 5 in Supplement 1).

## Discussion

In this population-level cohort study, we found that MHSA was associated with a significant reduction in risk of reincarceration. Prior studies have been mixed on this association. For example, 11 of 13 studies in a recent meta-analysis^29^ identified improved justice outcomes among people who accessed mental health and/or substance use services after release. Nevertheless, MHSA has been associated with an increased risk of reincarceration among people with severe mental illness.^30^ This has been attributed to mental health treatment serving as a form of community monitoring and surveillance, resulting in subsequent technical violations, particularly for people on parole.^31^

The mixed nature of results has also been attributed to the lack of examination of timeliness and intensiveness of MHSA after release, which remains a major gap in the literature.^31,32^ To our knowledge, our study is the first to quantify the association of timeliness of MHSA and subsequent reincarceration risk among people with mental disorder diagnoses released from prison. In this study, we found that for each additional month between release and services access, there was a 4% (statistically significant) increase in reincarceration risk. This emphasizes the need for timely services to reduce the risk of reincarceration, which can be supported by discharge planning beginning at the time of admission and continuing after release.^33^ Timeliness of services can also be facilitated by the expansion and implementation of peer-led services, which have been found to increase trust by meeting people where they are.^34,35^

We also examined associations with reincarceration by the type of mental health service accessed. We found that outpatient emergency services were associated with significantly higher risk of reincarceration compared with outpatient primary care visits. This emphasizes the pivotal role of regular contact with primary care services after release.^36^ This will require system interventions, including discharge planning to facilitate linkage to community services and to lessen the burden of competing postrelease priorities, such as securing housing and employment.^37^ Stigma is a known major barrier to engagement with primary care after release.^38^ Investment in peer-led interventions can play a critical role in rebuilding trust and bridging connections to care for people with mental disorders and/or SUDs who are transitioning from prison to community.^39,40,41,42,43^

In this study, we examined the associations of concurrent SUD and various mental disorder diagnoses with reincarceration risk. Our findings relating to differences by mental disorder diagnosis were consistent with those of other studies,^13,16^ in that we found that people in all diagnosis groups had a significantly lower hazard of MHSA compared with people with schizophrenia or other psychotic disorders. This could reflect the urgency of service need in this population, resulting in emergency department visits or hospitalizations, which are known to occur at elevated rates among people with severe mental disorder diagnoses.^44^ It could also reflect mandated contact with mental health services as a condition of release, which has been associated with reincarceration among people with the most severe mental disorder diagnoses.^21,31^ Although reincarceration risk was lower in all groups compared with those with schizophrenia or other psychotic disorders in the absence of MHSA (transition from state 1 to state 3), this association did not hold in the transition from MHSA to reincarceration (transition from state 2 to state 3). This suggests that MHSA is associated with reduced risk of reincarceration, even among people with the most severe mental disorder diagnoses.

Despite the association of MHSA and reincarceration, more than 40% of releases resulted in reincarceration without any MHSA, suggesting a high level of unmet health service needs in this population. Prior studies^45^ have similarly identified a high level of unmet mental health service needs among people with mental disorder diagnoses in North America. For example, a US study^46^ found that among people with serious mental disorder diagnoses, only 40% had received any treatment services in the prior year. These rates of access have been found to be even lower^31,47^ when considering minimally adequate care involving both clinical visits and medication access. There is, therefore, a need for future studies to investigate approaches to improving the accessibility of mental health services for people with mental disorder diagnoses released from prison.

### Limitations

There are a number of limitations in this study that must be considered. First, we relied on diagnostic codes from administrative health data to identify people with mental disorder diagnoses; therefore, people who were not in contact with health services were not captured in this analysis. Our sample represents people who spent at least 1 day in provincial custody during the study period and who were charged and/or convicted of an offense. Anyone who was only supervised in the community and never entered a prison is not included in the analysis. Furthermore, data on charge and conviction types (eg, property related or drug related) were not available in our data. Our data include only information on biological sex as a binary variable (male or female), with no information on gender identity, and data on race and ethnicity were not available. Our analysis accounts for participants’ first MHSA following release but does not account for the association of subsequent MHSA with reincarceration. Furthermore, we do not have data on MHSA during incarceration. We did not account for the quality of or appropriateness of services, which could be investigated in future studies using mixed quantitative and qualitative data.^48^ Furthermore, the findings may not be generalizable to other settings outside of BC, given that correctional settings and community mental health service availability vary by region.

## Conclusions

This cohort study found that MHSA was associated with a reduced hazard of reincarceration among people with mental disorder diagnoses released from prison in BC. We found that each additional month that passed between release and MHSA was associated with an increase in reincarceration risk, suggesting the importance of timely MHSA to reducing reincarceration. In BC, the mental health service needs of people released from prison remain largely unmet. Scaling up of timely access to mental health services after release is critical. This must be done with attention to the service needs of people who have concurrent SUD and to people with the most severe mental disorder diagnoses, who face the highest risk of reincarceration.

## Notes

Supplement 1. **eTable 1.** Mental Illness Diagnosis **eTable 2.** *ICD-9* and* ICD-10* Codes for Substance Use Disorder **eTable 3.** Comorbidities: *ICD-10 *Codes Used in Elixhauser Comorbidity Categories **eFigure 1.** Flowchart for Sample of 20% Random BC Population Who Were Released From Incarceration in a BC Provincial Correctional Centre Between January 1, 2015, and December 31, 2018, and Had a Mental Illness Diagnosis Prior to Release **eFigure 2.** Testing Linearity of MH Services Time and Hazard of Reincarceration Using a Smoothing Spline **eFigure 3.** Testing a Quadratic Term for the Relationship of MH Services Time and Hazard of Reincarceration **eFigure 4.** Determining Spline Degrees of Freedom Using AIC **eFigure 5.** State Occupation Probability Plots for a Reference Person, by Mental Disorder Type **eFigure 6.** State Occupation Probability Plots for a Reference Person, by SUD **eFigure 7.** Stratified Hazard Models to Verify the Proportional Hazard Assumption **eTable 4.** Reincarceration Rates With or Without Mental Disorder Diagnosis **eTable 5.** Hazard Ratios for Each of the 3 Transitions Estimated From Stratified Cox Proportional Hazards Models Among Releases With SUD Diagnosis Only (N=2,939)

Supplement 2.
Data Sharing Statement

## References

[1] Fazel S, Hayes AJ, Bartellas K, Clerici M, Trestman R. Mental health of prisoners: prevalence, adverse outcomes, and interventions. Lancet Psychiatry. 2016;3(9):871-881. doi:10.1016/S2215-0366(16)30142-0 27426440 PMC5008459

[2] Butler A, Nicholls T, Samji H, Fabian S, Lavergne MR. Prevalence of mental health needs, substance use and co-occurring disorders among people admitted to prison. Psychiatr Serv. 2022;73(7):737-744. doi:10.1176/appi.ps.202000927 34809437

[3] Fazel S, Seewald K. Severe mental illness in 33,588 prisoners worldwide: systematic review and meta-regression analysis. Br J Psychiatry. 2012;200(5):364-373. doi:10.1192/bjp.bp.111.096370 22550330

[4] Hamilton L, Belenko S. Effects of pre-release services on access to behavioral health treatment after release from prison. Justice Q. 2016;33(6):1080-1102. doi:10.1080/07418825.2015.1073771

[5] Lennox C, Senior J, King C, . The management of released prisoners with severe and enduring mental illness. J Forensic Psychiatry Psychol. 2012;23(1):37-41. doi:10.1080/14789949.2011.634921

[6] Baranyi G, Scholl C, Fazel S, Patel V, Priebe S, Mundt AP. Severe mental illness and substance use disorders in prisoners in low-income and middle-income countries: a systematic review and meta-analysis of prevalence studies. Lancet Glob Health. 2019;7(4):e461-e471. doi:10.1016/S2214-109X(18)30539-4 30879509 PMC6419715

[7] Ministry of Public Safety and Solicitor General. Profile of BC corrections. 2021. Accessed November 10, 2022. https://www2.gov.bc.ca/assets/gov/law-crime-and-justice/criminal-justice/corrections/reports-publications/bc-corrections-profile.pdf

[8] Hughes T, Wilson DJ. Re-entry trends in the United States. Bureau of Justice Statistics. 2002. Accessed November 7, 2022. https://bjs.ojp.gov/content/pub/pdf/reentry.pdf

[9] Keen C, Kinner SA, Young JT, . Periods of altered risk for non-fatal drug overdose: a self-controlled case series. Lancet Public Health. 2021;6(4):e249-e259. doi:10.1016/S2468-2667(21)00007-4 33773635

[10] Gan WQ, Kinner SA, Nicholls TL, . Risk of overdose-related death for people with a history of incarceration. Addiction. 2021;116(6):1460-1471. doi:10.1111/add.15293 33047844

[11] Binswanger IA, Stern MF, Deyo RA, . Release from prison: a high risk of death for former inmates. N Engl J Med. 2007;356(2):157-165. doi:10.1056/NEJMsa064115 17215533 PMC2836121

[12] Dong KR, Must A, Tang AM, Beckwith CG, Stopka TJ. Competing priorities that rival health in adults on probation in Rhode Island: substance use recovery, employment, housing, and food intake. BMC Public Health. 2018;18(1):289. doi:10.1186/s12889-018-5201-7 29482529 PMC5828298

[13] Baillargeon J, Penn JV, Knight K, Harzke AJ, Baillargeon G, Becker EA. Risk of reincarceration among prisoners with co-occurring severe mental illness and substance use disorders. Adm Policy Ment Health. 2010;37(4):367-374. doi:10.1007/s10488-009-0252-9 19847638

[14] Butler A, Nicholls T, Samji H, Fabian S, Lavergne MR. Prevalence of mental health needs, substance use, and co-occurring disorders among people admitted to prison. Psychiatr Serv. 2022;73(7):737-744. doi:10.1176/appi.ps.202000927 34809437

[15] British Columbia Justice and Public Safety Council. Performance measurement update for the justice and public safety sector. 2017. Accessed November 7, 2022. https://www.justicebc.ca/app/uploads/sites/11/2016/03/pm-nov-2015.pdf

[16] Zgoba KM, Reeves R, Tamburello A, Debilio L. Criminal recidivism in inmates with mental illness and substance use disorders. J Am Acad Psychiatry Law. 2020;48(2):209-215. doi:10.29158/JAAPL.003913-20 32051198

[17] Magee LA, Fortenberry JD, Rosenman M, Aalsma MC, Gharbi S, Wiehe SE. Two-year prevalence rates of mental health and substance use disorder diagnoses among repeat arrestees. Health Justice. 2021;9(1):2. doi:10.1186/s40352-020-00126-2 33411067 PMC7789256

[18] Jones RM, Manetsch M, Gerritsen C, Simpson AIF. Patterns and predictors of reincarceration among prisoners with serious mental illness: a cohort study. Can J Psychiatry. 2021;66(6):560-568. doi:10.1177/0706743720970829 33155829 PMC8138736

[19] Van Dorn RA, Desmarais SL, Petrila J, Haynes D, Singh JP. Effects of outpatient treatment on risk of arrest of adults with serious mental illness and associated costs. Psychiatr Serv. 2013;64(9):856-862. doi:10.1176/appi.ps.201200406 23677480

[20] Wilson AB, Draine J, Hadley T, Metraux S, Evans A. Examining the impact of mental illness and substance use on recidivism in a county jail. Int J Law Psychiatry. 2011;34(4):264-268. doi:10.1016/j.ijlp.2011.07.004 21839518

[21] Hopkin G, Evans-Lacko S, Forrester A, Shaw J, Thornicroft G. Interventions at the transition from prison to the community for prisoners with mental illness: a systematic review. Adm Policy Ment Health. 2018;45(4):623-634. doi:10.1007/s10488-018-0848-z 29362981 PMC5999162

[22] Xavier C, Zhao B, Gan WQ, Slaunwhite A. Provincial overdose cohort: population data linkage during an overdose crisis. Int J Popul Data Sci. 2020;5(5). doi:10.23889/ijpds.v5i5.1468

[23] Keen C, Kinner SA, Young JT, . Prevalence of co-occurring mental illness and substance use disorder and association with overdose: a linked data cohort study among residents of British Columbia, Canada. Addiction. 2022;117(1):129-140. doi:10.1111/add.15580 34033179

[24] BC Ministry of Health. Consolidation file (MSP registration and premium billing) data extract. Accessed November 10, 2022. https://www.popdata.bc.ca/data/demographic/consolidation_file

[25] British Columbia Ministry of Health DADHS. National ambulatory care reporting system. 2018. Accessed November 10, 2022. https://www2.gov.bc.ca/assets/gov/health/forms/5454datadictionary.pdf

[26] British Columbia Ministry of Health DE. Discharge abstracts database. 2018. Accessed November 10, 2022. https://www.popdata.bc.ca/data/health/dad

[27] Elixhauser A, Steiner C, Harris DR, Coffey RM. Comorbidity measures for use with administrative data. Med Care. 1998;36(1):8-27. doi:10.1097/00005650-199801000-00004 9431328

[28] Therneau TM, Grambsch PM. Modeling Survival Data: Extending the Cox Model. Springer; 2000.

[29] Stewart AC, Cossar RD, Quinn B, . Criminal justice involvement after release from prison following exposure to community mental health services among people who use illicit drugs and have mental illness: a systematic review. J Urban Health. 2022;99(4):635-654. doi:10.1007/s11524-022-00635-5 35501591 PMC9360359

[30] Calsyn RJ, Yonker RD, Lemming MR, Morse GA, Klinkenberg WD. Impact of assertive community treatment and client characteristics on criminal justice outcomes in dual disorder homeless individuals. Crim Behav Ment Health. 2005;15(4):236-248. doi:10.1002/cbm.24 16575844

[31] Domino ME, Gertner A, Grabert B, Cuddeback GS, Childers T, Morrissey JP. Do timely mental health services reduce re-incarceration among prison releasees with severe mental illness? Health Serv Res. 2019;54(3):592-602. doi:10.1111/1475-6773.13128 30829406 PMC6505414

[32] Barrenger S, Kriegel L, Canada K, Wilson A. What gets measured in reentry research? a scoping review on community reentry from jail and prison for persons with mental illnesses. Crim Justice Behav. 2021;48(3):259-273. doi:10.1177/0093854820983844

[33] La Vigne N, Davies E, Palmer T, Halberstadt R. Release Planning for Successful Reentry. Urban Institute Justice Policy Centre; 2008.

[34] Portillo S, Goldberg V, Taxman FS. Mental health peer navigators: working with justice-involved populations. Prison J. 2017;97:318-341. doi:10.1177/0032885517704001

[35] Perry AE, Waterman MG, Dale V, Moore K, House A. The effect of a peer-led problem-support mentor intervention on self-harm and violence in prison: an interrupted time series analysis using routinely collected prison data. EClinicalMedicine. 2021;32:100702. doi:10.1016/j.eclinm.2020.100702 33681733 PMC7910675

[36] Kinner SA, Young JT, Carroll M. The pivotal role of primary care in meeting the health needs of people recently released from prison. Australas Psychiatry. 2015;23(6):650-653. doi:10.1177/1039856215613008 26498149

[37] Fox AD, Anderson MR, Bartlett G, Valverde J, Starrels JL, Cunningham CO. Health outcomes and retention in care following release from prison for patients of an urban post-incarceration transitions clinic. J Health Care Poor Underserved. 2014;25(3):1139-1152. doi:10.1353/hpu.2014.0139 25130230 PMC4138598

[38] Martin K, Taylor A, Howell B, Fox A. Does criminal justice stigma affect health and health care utilization? a systematic review of public health and medical literature. Int J Prison Health. 2020;16(3):263-279. doi:10.1108/IJPH-01-2020-0005 33634660 PMC11016312

[39] McLeod KE, Timler K, Korchinski M, . Supporting people leaving prisons during COVID-19: perspectives from peer health mentors. Int J Prison Health. 2021;17(3):206-216. doi:10.1108/IJPH-09-2020-0069 33656310 PMC8753623

[40] McLeod KE, Korchinski M, Young P, . Supporting women leaving prison through peer health mentoring: a participatory health research study. CMAJ Open. 2020;8(1):E1-E8. doi:10.9778/cmajo.20190106 32071141 PMC7028165

[41] Russell C, Nafeh F, Pang M, . Opioid agonist treatment (OAT) experiences and release plans among federally incarcerated individuals with opioid use disorder (OUD) in Ontario, Canada: a mixed-methods study. BMC Public Health. 2022;22(1):436. doi:10.1186/s12889-022-12685-0 35246083 PMC8897889

[42] Shavit S, Aminawung JA, Birnbaum N, . Transitions clinic network: challenges and lessons in primary care for people released from prison. Health Aff (Millwood). 2017;36(6):1006-1015. doi:10.1377/hlthaff.2017.0089 28583958

[43] Kendall S, Redshaw S, Ward S, Wayland S, Sullivan E. Systematic review of qualitative evaluations of reentry programs addressing problematic drug use and mental health disorders amongst people transitioning from prison to communities. Health Justice. 2018;6(1):4. doi:10.1186/s40352-018-0063-8 29500640 PMC5834412

[44] Snow KJ, Petrie D, Young JT, Preen DB, Heffernan E, Kinner SA. Impact of dual diagnosis on healthcare and criminal justice costs after release from Queensland prisons: a prospective cohort study. Aust J Prim Health. 2022;28(3):264-270. doi:10.1071/PY21142 35512815

[45] Smith JL, Khatri UG, Olubiyi O, . Behavioral health service use post-jail release and reduced risk of return to jail. J Community Psychol. 2022;50(7):3044-3053. doi:10.1002/jcop.22813 35132631

[46] Wang EA, Lin HJ, Aminawung JA, . Propensity-matched study of enhanced primary care on contact with the criminal justice system among individuals recently released from prison to New Haven. BMJ Open. 2019;9(5):e028097. doi:10.1136/bmjopen-2018-028097 31048315 PMC6502013

[47] Wang EA, Hong CS, Shavit S, Sanders R, Kessell E, Kushel MB. Engaging individuals recently released from prison into primary care: a randomized trial. Am J Public Health. 2012;102(9):e22-e29. doi:10.2105/AJPH.2012.300894 22813476 PMC3482056

[48] Reardon T, Harvey K, Baranowska M, O’Brien D, Smith L, Creswell C. What do parents perceive are the barriers and facilitators to accessing psychological treatment for mental health problems in children and adolescents? a systematic review of qualitative and quantitative studies. Eur Child Adolesc Psychiatry. 2017;26(6):623-647. doi:10.1007/s00787-016-0930-6 28054223 PMC5446558

