# Supplementary material for: Association of Mental Health Services Access and Reincarceration Among Adults Released From Prison in British Columbia, Canada
Source: JAMA Netw Open. 2022 Dec 15;5(12):e2247146. doi: 10.1001/jamanetworkopen.2022.47146 (PMC9856264; doi:10.1001/jamanetworkopen.2022.47146)
Supplement: Supplement 1. — eTable 1. Mental Illness Diagnosis eTable 2. ICD-9 and ICD-10 Codes for Substance Use Disorder eTable 3. Comorbidities: ICD-10 Codes Used in Elixhauser Comorbidity Categories eFigure 1. Flowchart for Sample of 20% Random BC Population Who Were Released From Incarceration in a BC Provincial Correctional Centre Between January 1, 2015, and December 31, 2018, and Had a Mental Illness Diagnosis Prior to Release eFigure 2. Testing Linearity of MH Services Time and Hazard of Reincarceration Using a Smoothing Spline eFigure 3. Testing a Quadratic Term for the Relationship of MH Services Time and Hazard of Reincarceration eFigure 4. Determining Spline Degrees of Freedom Using AIC eFigure 5. State Occupation Probability Plots for a Reference Person, by Mental Disorder Type eFigure 6. State Occupation Probability Plots for a Reference Person, by SUD eFigure 7. Stratified Hazard Models to Verify the Proportional Hazard Assumption eTable 4. Reincarceration Rates With or Without Mental Disorder Diagnosis eTable 5. Hazard Ratios for Each of the 3 Transitions Estimated From Stratified Cox Proportional Hazards Models Among Releases With SUD Diagnosis Only (N=2,939) [file jamanetwopen-e2247146-s001.pdf]

Supplemental Online Content

Palis H, Hu K, Rioux W, et al. Association of mental health services access and reincarceration among adults released from prison in British Columbia, Canada. *JAMA Netw Open*. 2022;5(12):e2247146. doi:10.1001/jamanetworkopen.2022.47146

eTable 1. Mental Illness Diagnosis
eTable 2. *ICD-9* and *ICD-10* Codes for Substance Use Disorder
eTable 3. Comorbidities: *ICD-10* Codes Used in Elixhauser Comorbidity Categories
eFigure 1. Flowchart for Sample of 20% Random BC Population Who Were Released From Incarceration in a BC Provincial Correctional Centre Between January 1, 2015, and December 31, 2018, and Had a Mental Illness Diagnosis Prior to Release
eFigure 2. Testing Linearity of MH Services Time and Hazard of Reincarceration Using a Smoothing Spline
eFigure 3. Testing a Quadratic Term for the Relationship of MH Services Time and Hazard of Reincarceration
eFigure 4. Determining Spline Degrees of Freedom Using AIC
eFigure 5. State Occupation Probability Plots for a Reference Person, by Mental Disorder Type
eFigure 6. State Occupation Probability Plots for a Reference Person, by SUD
eFigure 7. Stratified Hazard Models to Verify the Proportional Hazard Assumption
eTable 4. Reincarceration Rates With or Without Mental Disorder Diagnosis
eTable 5. Hazard Ratios for Each of the 3 Transitions Estimated From Stratified Cox Proportional Hazards Models Among Releases With SUD Diagnosis Only (N=2,939)

This supplemental material has been provided by the authors to give readers additional information about their work.

© 2022 Palis H et al. *JAMA Network Open.*

eTable 1: Mental Illness Diagnosis

	Hospital (ICD-10)	Primary Care (ICD-9)	Group reflected in analysis
**Mental illness**			
1. Neurocognitive disorders	F00-F09	290, 293, 294, 310	Other
2. Schizophrenia spectrum disorders and other psychotic disorders	F20-F29	295, 297, 298,	Schizophrenia
3. Mood [affective] disorders	F30-F39	296, 3004, 311	Mood disorders
4. Neurotic, stress-related and somatoform disorders	F40-F48	300 (minus 3004), 306, 3078, 308, 309	Stress related disorders
5. Behavioural syndromes associated with physiological disturbances and physical factors	F50-F59	3027, 3071, 3074, 3075, 316	Personality or behavioural disorders
6. Disorders of adult personality and behaviour	F60-F69	301, 302 (minus 3027), 3079, 3123	Personality or behavioural disorders
7. Intellectual disabilities	F70-F79	317, 318, 319	Other
8. Disorders of psychological development	F80-F89	299, 315	Other
9. Behavioural and emotional disorders with onset usually occurring in childhood and adolescence	F90-F98	307 (minus 3071, 3074, 3075, 3078, 3079), 312 (minus 3123), 313, 314	Personality or behavioural disorders
10. Multiple, other, or unspecified mental illness	F99	50B	Other

Footnote: Mental disorder diagnosis was determined to be present for people with one hospitalization or two primary care visits with a relevant ICD9/10 code (See Supplement Table S1) within 1 year of each other, and occurring in the 1 year prior to their release (20).

eTable 2: *ICD-9* and *ICD-10* Codes for Substance Use Disorder

Substance use disorder	Hospital (*ICD-10*)	Primary Care (*ICD-9*)
1. Alcohol	F10	291, 303, 3050
2. Opioids	F11	3040, 3055
3. Cannabinoids	F12	3043, 3052
4. Sedatives or hypnotics	F13	3041, 3054
5. Cocaine	F14	3042, 3056
6. Stimulants	F15	3044, 3057
7. Hallucinogens	F16	3045, 3053
8. Tobacco	F17	3051
9. Solvents	F18	3059
10. Multiple, other, and unspecified psychoactive substances	F19	292, 3047, 3048, 3049, 3058

© 2022 Palis H et al. *JAMA Network Open.*

eTable 3: Comorbidities: *ICD-10* Codes Used in Elixhauser Comorbidity Categories

Category	*ICD-10* codes
1. Congestive heart failure	I099, I110, I130, I193, I255, I420, I425, I426, I427, I428, I429, I43, I50, P290
2. Cardiac arrhythmia	I441, I442, I443, I456, I459, I47, I48, I49, R000, R001, R008, T821, Z450, Z950
3. Valvular disease	A520, I05, I06, I07, I08, I091, I098, I34, I35, I36, I37, I38, I39, Q231, Q232, Q233, Z952, Z953, Z954
4. Pulmonary circulation disorders	I26, I27, I280, I288, I289
5. Peripheral vascular disorders	I70, I71, I731, I738, I739, I771, I790, I792, K551, K558, K559, Z958, Z959
6. Hypertension uncomplicated	I10
7. Hypertension complicated	I11, I12, I13, I15
8. Paralysis	G041, G114, G801, G802, G81, G82, G830, G831, G832, G833, G834, G839
9. Other neurological disorders	G10, G11, G12, G13, G20, G21, G22, G254, G255, G312, G318, G319, G32, G35, G36, G37, G40, G41, G931, G934, R470, R56
10. Chronic pulmonary disease	I278, I279, J40, J41, J43, J44, J45, J46, J47, J60, J61, J62, J63, J64, J65, J66, J67, J684, J701, J703
11. Diabetes uncomplicated	E100, E101, E109, E110, E111, E119, E120, E121, E129, E130, E131, E139, E140, E141, E149
12. Diabetes complicated	E10-E0E108, E112-E118, E122-E128, E132-E138, E14-E148
13. Hypothyroidism	E00-E03, E890
14. Renal Failure	I120, I131, N18, N19, N250, Z490, Z491, Z492, Z940, Z992
15. Liver disease	B18, I85, I865, I982, K70, K711, K713, K714, K715, K717, K72, K73, K74, K760, K762-K769, Z94
16. Peptic ulcer excluding bleeding	K257, K259, K267, K269, K277, K279, K287, K289
17. HIV/AIDS	B20, B21, B22, B24
18. Lymphoma	C81-C85, C88, C96, C900, C902
19. Metastic cancer	C77-C80
20. Solid tumor without metastasis	C00-C26, C30-C34, C37-C41, C43, C45-C58, C60-C76, C97
21. Rheumatoid arthritis/collagen	L940, L941, L943, M05, M06, M08, M120, M123, M30, M310, M311, M312, M313, M32, M33, M34, M35, M45, M461, M468, M469
22. Coagulopathy	D65, D66, D67, D68, D691, D693, D694, D695, D696
23. Obesity	E66
24. Weight loss	E40-E46, R634, R64
25. Fluid and electrolyte disorders	E222, E86, E87
26. Blood loss anemia	D500
27. Deficiency anemia	D508, D509, D51, D52, D53

Based on ICD-10 codes categorised by Quan et al[1]

[1] Quan, H., Sundararajan, V., Halfon, P., Fong, A., Burnand, B., Luthi, J. C., ... & Ghali, W. A. (2005). Coding algorithms for defining comorbidities in ICD-9-CM and ICD-10 administrative data. *Medical care*, 1130-1139

© 2022 Palis H et al. *JAMA Network Open.*

eFigure 1: Flowchart for Sample of 20% Random BC Population Who Were Released From Incarceration in a BC Provincial Correctional Centre Between January 1, 2015, and December 31, 2018, and Had a Mental Illness Diagnosis Prior to Release

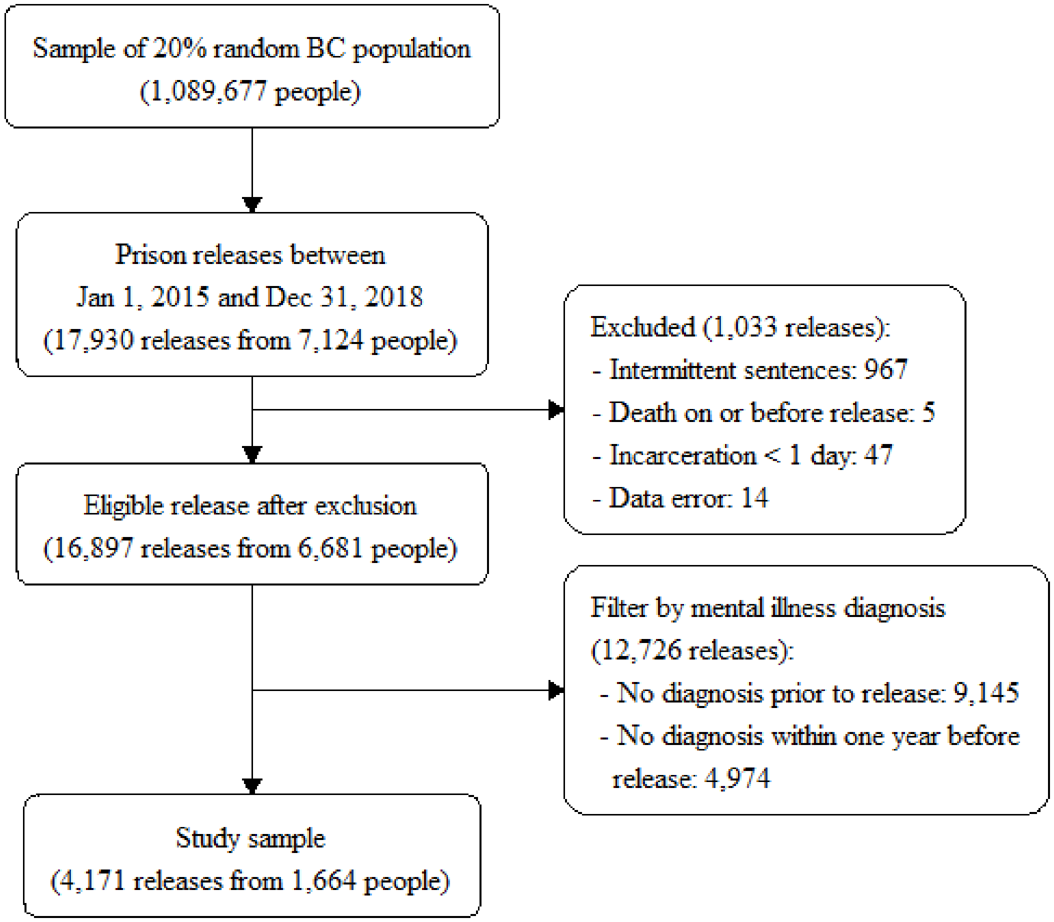

© 2022 Palis H et al. *JAMA Network Open.*

eFigure 2: Testing Linearity of MH Services Time and Hazard of Reincarceration Using a Smoothing Spline

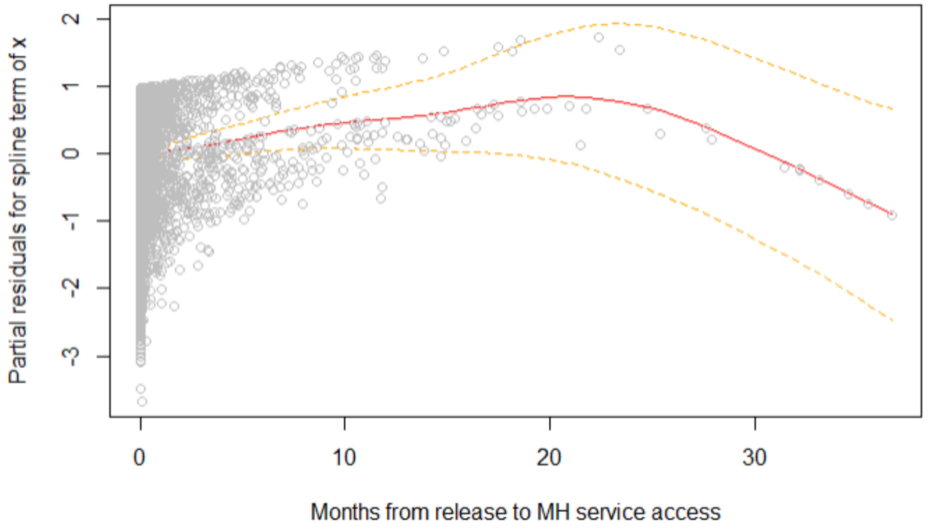

Footnote: Smoothing spline approach was tested following Therneau & Grambsch, 2000 (Modeling Survival Data, p107-111). We can see that the log hazard increase over the first 2 years then decrease sharply. However, the confidence intervals of the decrease are wide and are caused by only a few observations (the grey dots represent partial residuals; there are very few observations that MH time > 20 (N=16= 0.8% of all 1927 2→3 transitions)).

eFigure 3: Testing a Quadratic Term for the Relationship of MH Services Time and Hazard of Reincarceration

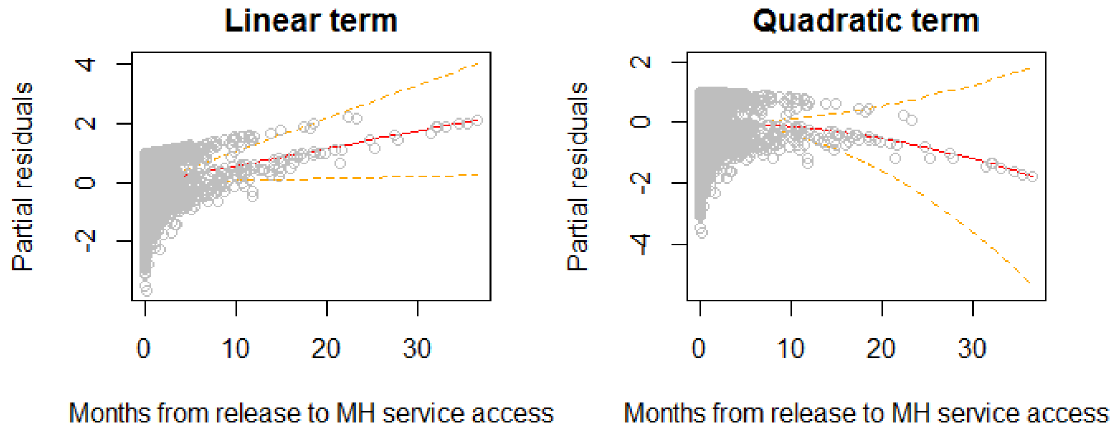

Footnote: In an alternative model with an additional quadratic term of MH service time, the quadratic term acts as an adjustment for large MH time values. However, the quadratic term is not statistically significant: hazard ratios for the linear and quadratic terms were estimated to be 1.06 (CI: 1.01, 1.12; p-value: 0.023) and 1.00 (CI: 0.996, 1.001; p-value: 0.329), respectively.

© 2022 Palis H et al. *JAMA Network Open.*

eFigure 4: Determining Spline Degrees of Freedom Using AIC

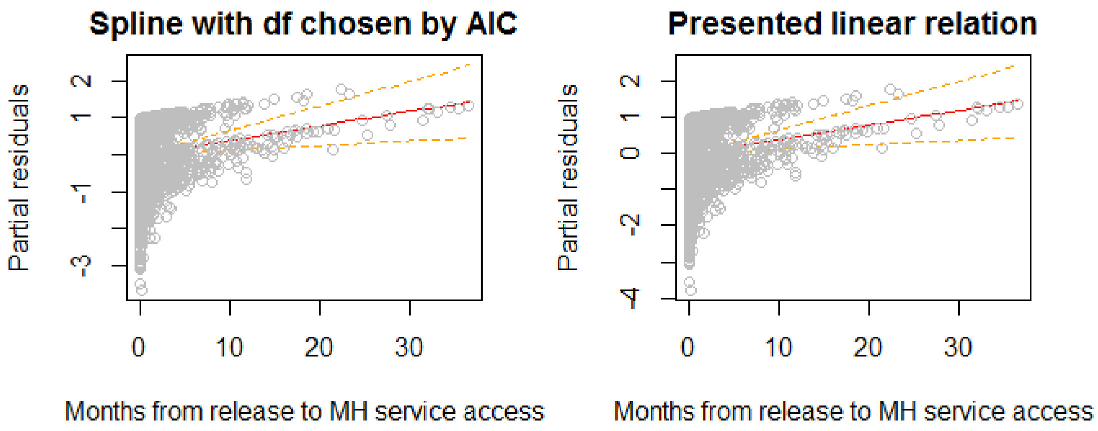

Footnote: When using AIC to choose the degree of freedom of the spline (i.e., how many segments the spline has), the result is a straight line (figure on the left) suggesting a linear relation (matching with the linear relation in the model reflected in the figure on the right).

© 2022 Palis H et al. *JAMA Network Open.*

eFigure 5: State Occupation Probability Plots for a Reference Person, by Mental Disorder Type

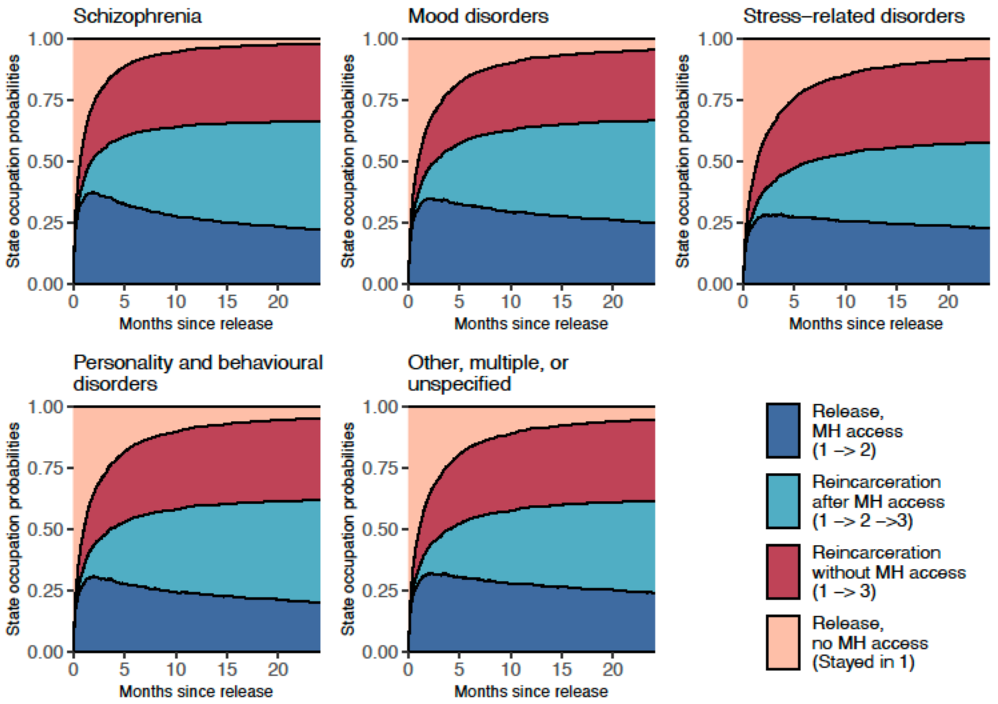

Footnote*:* All other variables held constant as follows: sex=male, HA= Vancouver coastal, MH SUD= no, age=30-39, sdpr=no, elix=0, in-prison time = 16-60 days

© 2022 Palis H et al. *JAMA Network Open.*

eFigure 6: State Occupation Probability Plots for a Reference Person, by SUD

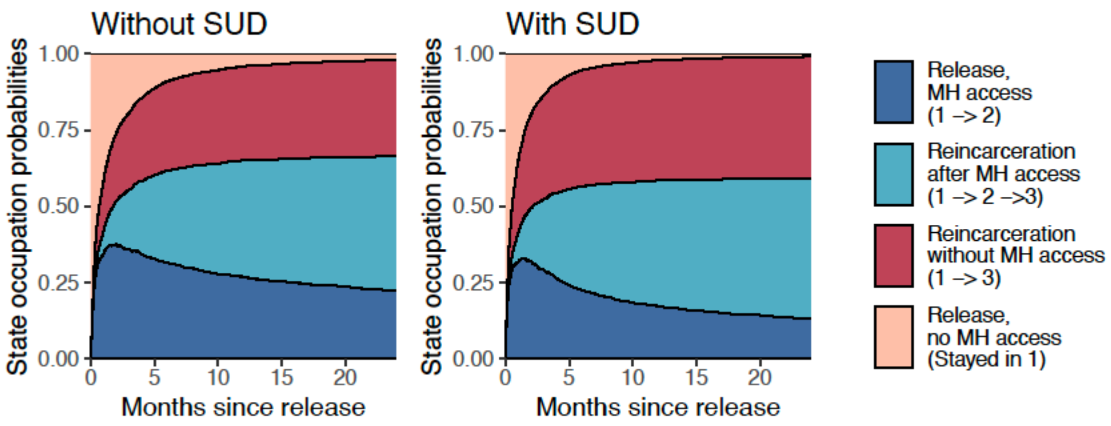

Footnote: All other variables held constant as follows: sex=male, HA= Vancouver coastal, MH class= schizophrenia, age=30-39, sdpr=no, elix=0, in-prison time = 16-60 days

eFigure 7: Stratified Hazard Models to Verify the Proportional Hazard Assumption

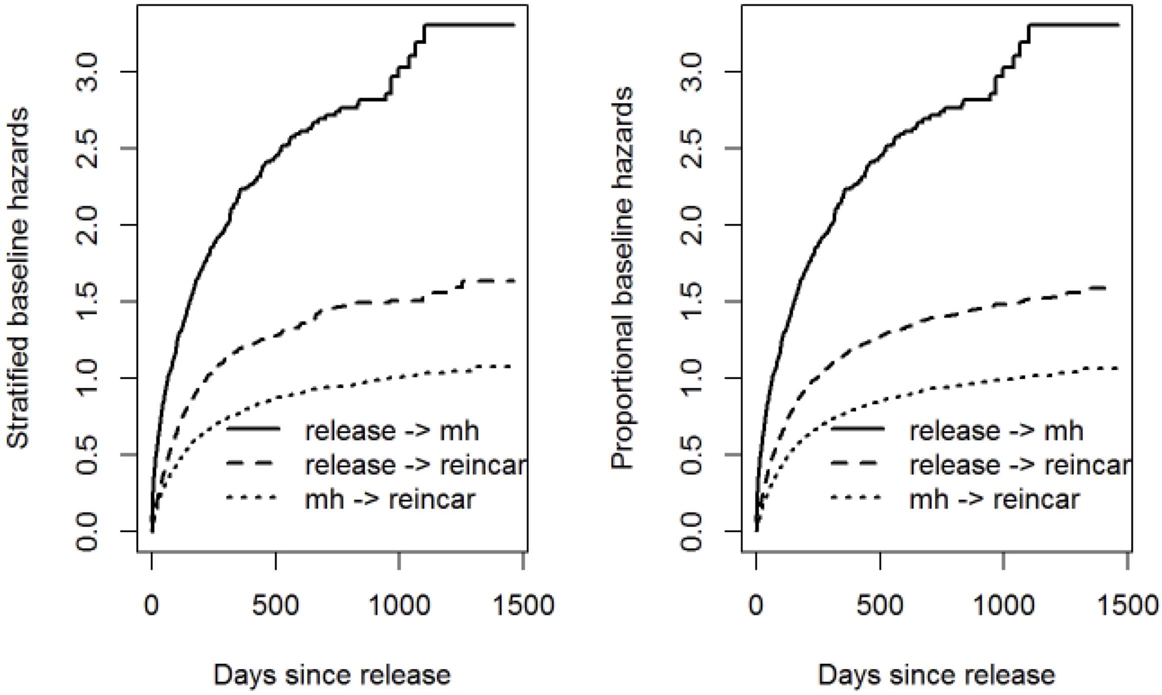

Footnote: the shapes of the two transitions to reincarceration in the stratified baseline hazards are similar to in the proportional baseline hazards model. Therefore it is reasonable to assume proportional hazards.

© 2022 Palis H et al. *JAMA Network Open.*

eTable 4: Reincarceration Rates With or Without Mental Disorder Diagnosis

		Mental disorder diagnosis			
Characteristic	Overall, N = 6,681[a]	Never, N = 4,033[a]	Not recent, N = 984[a]	Recent, N = 1,664[a]	p-value[b]
**Reincarceration**					<0.001
No	3,467 (51.9)	2,401 (59.5)	536 (54.5)	530 (31.9)	
Yes	3,214 (48.1)	1,632 (40.5)	448 (45.5)	1,134 (68.1)	
[a]n (%)					
[b]Pearson’s Chi-squared test					

Footnote: Recent= in the one year prior to release; Not recent= earlier than one year prior to release

© 2022 Palis H et al. *JAMA Network Open.*

eTable 5: Hazard Ratios for Each of the 3 Transitions Estimated From Stratified Cox Proportional Hazards Models Among Releases With SUD Diagnosis Only (N=2,939)

	**Release -ReInc 1→3**	**Release –MH Srv 1→2**	**MH Srv – ReInc 2→3**
**Receiving state stratum** [a]	**State 3**	**State 2**	**State 3**
**Variable**	**HR 95% CI**	**HR 95% CI**	**HR 95% CI**
**Age group**			
<30	Reference	Reference	Reference
30-39	0.95(0.81-1.12)	0.95(0.80-1.13)	0.98(0.78-1.23)
40-49	0.90(0.73-1.11)	0.94(0.77-1.15)	0.81(0.64-1.02)
>= 50	0.56(0.40-0.78)	1.03(0.78-1.34)	0.61(0.42-0.87)
**Sex**			
Female	Reference	Reference	Reference
Male	1.43(1.16-1.75)	1.03(0.86-1.23)	1.27(1.05-1.55)
**Health Authority**			
Fraser	Reference	Reference	Reference
Interior	0.57(0.43-0.75)	1.01(0.80-1.27)	1.08(0.84-1.40)
Northern	0.79(0.56-1.10)	0.80(0.62-1.04)	0.73(0.53-1.01)
Vancouver Coastal	0.94(0.77-1.14)	1.08(0.89-1.31)	1.22(0.91-1.64)
Vancouver Island	0.82(0.65-1.04)	0.86(0.68-1.08)	0.89(0.68-1.18)
Unknown	1.03(0.78-1.34)	0.09(0.05-0.14)	0.30(0.08-1.13)
**Elixhauser comorbidity Index**			
0	Reference	Reference	Reference
1	1.12(0.86-1.46)	1.27(1.00-1.63)	1.15(0.78-1.68)
>=2	0.98(0.70-1.39)	1.06(0.85-1.33)	0.88(0.64-1.23)
**SA in prior year**			
No	Reference	Reference	Reference
Yes	1.45(1.20-1.75)	1.35(1.16-1.57)	1.44(1.19-1.74)
**Sentence length**			
< 4 days	Reference	Reference	Reference
4-15 days	0.86(0.73-1.01)	0.96(0.83-1.10)	1.14(0.91-1.43)

© 2022 Palis H et al. *JAMA Network Open.*

	**Release -ReInc 1→3**	**Release –MH Srv 1→2**	**MH Srv – ReInc 2→3**
16-60 days	0.91(0.77-1.07)	1.06(0.92-1.23)	1.18(0.96-1.46)
> 60 days	0.52(0.42-0.63)	0.97(0.81-1.18)	0.89(0.70-1.14)
**Release year**			
2015	Reference	Reference	Reference
2016	1.01(0.86-1.18)	1.01(0.86-1.18)	1.01(0.82-1.23)
2017	1.00(0.83-1.20)	0.96(0.81-1.14)	0.98(0.80-1.21)
2018	0.83(0.68-1.01)	0.93(0.77-1.11)	0.76(0.57-1.01)
**MH diagnosis**			
Schizophrenia	Reference	Reference	Reference
Mood disorder	0.78(0.64-0.94)	0.88(0.73-1.06)	1.03(0.84-1.26)
Stress related disorder	0.80(0.66-0.96)	0.72(0.60-0.87)	1.02(0.82-1.27)
Personality and behavioural disorders	0.78(0.60-1.03)	0.82(0.65-1.05)	0.99(0.74-1.31)
Other disorders [b]	0.83(0.64-1.07)	0.88(0.72-1.08)	1.03(0.82-1.29)
**MH services access (Yes)** [c]	-	-	0.53(0.34-0.85)
**Time to MH Services access (months)** [c]	-	-	1.03(1.00-1.07)
**MH services access type**			
Outpatient primary care	Reference	Reference	Reference
Outpatient emergency care	-	-	1.33(0.99-1.77)
Counselling	-	-	0.97(0.77-1.22)
Outpatient specialist care	-	-	1.13(0.84-1.52)
Hospitalization	-	-	1.32(1.00-1.73)
ED visit	-	-	1.27(0.82-1.96)
Other [d]	-	-	1.73(1.26-3.38)
**SUD service access**			
No	Reference	Reference	Reference
Yes	0.33(0.29-0.37)	0.49(0.43-0.55)	0.46(0.39-0.56)

Footnote: Abbreviations**:** ReInc= Reincarceration; MH Srv= MH services; SA= social assistance; SUD= substance use disorder; MH= mental health; HR= hazard ratio; CI= confidence interval

© 2022 Palis H et al. *JAMA Network Open.*

[a] Transitions 1→3 and 2→3 are in one stratum and share a common baseline hazard (receiving State 3), transition 1→2 is in a separate stratum (receiving state 2).
[b]Other disorders= Neurocognitive disorders, Intellectual disabilities, disorders of psychological development, and multiple, other or unspecified mental disorder. See supplement for further information.
[c]The association of MH access and timeliness with the outcome are estimated to be HR = 0.65(p-value = 0.044) and HR = 1.04(p-value = 0.004), respectively.
[d] Other includes the following service codes: “miscellaneous and other visits”; “institutional visits,”; “No charge referral”; “Consultation”; “Minor surgery or other procedure”; “Home visits”; “APB Encounters”; “Visit premiums” ; “Pathology

© 2022 Palis H et al. *JAMA Network Open.*
