# Supplementary material for: Association of Mental Health Services Access and Reincarceration Among Adults Released From Prison in British Columbia, Canada
Source: JAMA Netw Open. 2022 Dec 15;5(12):e2247146. doi: 10.1001/jamanetworkopen.2022.47146 (PMC9856264; doi:10.1001/jamanetworkopen.2022.47146)
Supplement: Supplement 2. — Data Sharing Statement [file jamanetwopen-e2247146-s002.pdf]

Data Sharing Statement

Palis. Association of Mental Health Services Access and Reincarceration Among Adults Released From Prison. *JAMA Netw Open*. Published December 15, 2022. doi:10.1001/jamanetworkopen.2022.47146

Data
Data available: No

Additional Information
Explanation for why data not available: Researchers can access the Provincial Overdose Cohort data through a proposal process submitted to Chloe.xavier@bccdc.ca. The Review Committee for the Cohort will consider proposals that support BC's response to the overdose public health emergency, with an emphasis on projects that address provincial and Cohort priorities.
